# HAG (Homoharringtonine, Cytarabine, G-CSF) Regimen for the Treatment of Acute Myeloid Leukemia and Myelodysplastic Syndrome: A Meta-Analysis with 2,314 Participants

**DOI:** 10.1371/journal.pone.0164238

**Published:** 2016-10-05

**Authors:** Mixue Xie, Qi Jiang, Li Li, Jingjing Zhu, Lixia Zhu, De Zhou, Yanlong Zheng, Xiudi Yang, Mingyu Zhu, Jianai Sun, Wanzhuo Xie, Xiujin Ye

**Affiliations:** 1 Senior Department of Haematology, The First Affiliated Hospital, College of Medicine, Zhejiang University, Hangzhou, Zhejiang Province, 310003, China; 2 Department of Medical Oncology, The First Affiliated Hospital, College of Medicine, Zhejiang University, Hangzhou, Zhejiang Province, 310003, China; Queen's University Belfast, UNITED KINGDOM

## Abstract

**Background:**

In China, the combination of homoharringtonine, cytarabine, and G-CSF (HAG) has been extensively applied for treatment of acute myeloid leukemia (AML) and myelodysplastic syndrome (MDS).

**Methods:**

We performed a meta-analysis of 2,314 patients (AML, n = 1754; MDS, n = 560) to determine the overall safety and efficacy of this regimen.

**Results:**

The complete response (CR) rate of AML patients (53%) was significantly higher than that of MDS/transformed-AML patients (45%; *P* = 0.007). The CR rate of patients with newly diagnosed AML (62%) was significantly higher than in patients with relapsed/refractory AML (50%; *P* = 0.001). There were no significant difference in CR rates between elderly AML patients (54%) and all AML patients (*P* = 0.721). When compared with non-HAG regimens for AML/MDS induction therapy, the CR rate of patients treated with HAG was significantly higher than in treated with intensive chemotherapy (*P* = 0.000). No significant differences in CR rates were observed between patients treated with HAG and those treated with CAG (cytarabine, aclarubicin, G-CSF) regimens (*P* = 0.073). HAG regimen was well tolerated, with early death (ED) in 2%, grade IV myelosurrpression in 52% and infection in 50%. Reports of ED and rates of myelosuppression were reduced as compared with intensive chemotherapy (*P* = 0.000 and *P* = 0.000, respectively).

**Conclusion:**

The HAG regimen is an effective and safe regimen for the treatment of AML and MDS, and appears to be more effective and better tolerated than intensive chemotherapy. Future randomized controlled trials and further meta-analyses are strongly needed to confirm its efficacy and safety, especially in comparison with intensive chemotherapy.

## Introduction

Standard induction therapy usually includes an anthracycline such as daunorubicin (Dau), idarubicin (Ida), or the anthracenedione mitoxantrone and cytarabine (Ara-C), which has resulted in improved treatment outcomes and prognosis for younger adults (<60 years) with acute myeloid leukemia (AML) [1]; however, in individuals with high-risk AML, including elderly, relapsed, refractory, and secondary AML, outcomes from intensive chemotherapy remain suboptimal. Biological characteristics contributing to these unsatisfactory outcomes include poor performance status (PS), poor tolerance for therapy, the emergence of drug resistant malignant clones, an immunocompromised state and multiple organ dysfunction. In 1995, Yamada and colleagues first proposed a novel low-dose chemotherapy for the treatment of AML consisting of low-dose cytarabine and aclarubicin combined with granulocyte colony-stimulating factor (G-CSF) priming, referred to as CAG [2]. The CAG regimen has been widely used in China and Japan for the past two decades and proven efficacious in the treatment of refractory and relapsed AML as well as high-risk myelodysplastic syndrome (MDS) [3]. However, the cardiac toxicity associated with aclarubicin limits to a certain extent the application of the CAG regimen, especially in elderly patients with cardiac disease.

A new chemotherapy regimen with less cardiac toxicity was developed for the treatment of AML consisting of low-dose homoharringtonine (HHT) and cytarabine as well as G-CSF priming, abbreviated as HAG. HHT is a plant alkaloid first isolated from *Cephalotaxus* in China and has been used successfully in the treatment of acute and chronic myeloid leukemia since the 1970s [4,5]. HHT is a protein synthesis inhibitor that causes leukemic cells to arrest at the G1/G2 phase of the cell cycle [6,7] and can induce dose-dependent apoptosis in many acute myeloid cell lines and primary myeloid leukemia cells [6,8–9]. Cytarabine acts at the S-phase of the cell cycle to induce apoptosis. HHT and cytarabine act in a synergistic manner to induce apoptosis of leukemic cells. G-CSF leads to an enrichment of S-phase leukemic blasts [10], thereby improving the efficacy of cytarabine, which is active in the S-phase.

A significant number of clinical trials have been performed using the HAG regimen in China. The majority of these studies are published in Chinese, limiting the availability of these articles to the global scientific community. To provide an overview of these results, we performed a literature search and meta-analysis of 2,314 patients from fifty-six studies to assess the overall efficacy and safety of HAG regimen in AML and MDS patients.

## Design and Methods

### Data Sources

Databases including PubMed, EMBASE, China National Knowledge Infrastructure, Wanfang Data, and the American Society of Hematology (ASH) meeting abstracts were searched for articles published in English or Chinese between January 2005 and December 2014. Eligible studies were relevant clinical trials of AML or MDS patients treated with HAG. Key words used in the search were “homoharringtonine”, “cytarabine”, “G-CSF”, “HAG”,“CHG”, “myelodysplastic syndrome”, “MDS”, “acute myeloid leukemia” and “AML”. This meta-analysis was performed in accordance with the Preferred Reporting Items for Meta-Analyses (PRISMA) statement checklist (S1 Checklist).

### Study Selection, Meta-analysis Inclusion Criteria, and Data Extraction

The publications selected for inclusion were carefully screened. Preclinical studies, case reports and reviews were excluded. All publications identified based on our inclusion criteria were screened by two reviewers (Mixue Xie, Qi Jiang). In the event of disagreement between the two reviewers, we obtained and independently inspected the full text article. In total, 56 studies were chosen for the final analysis.

Inclusion criteria for studies in the meta-analysis were: (1) a minimum of 20 patients with MDS or AML; (2) treatment with the HAG regimen, and without additional chemotherapy,immunotherapy, epigenetic therapy or hematopoietic stem cell transplantation; (3)reported in English or Chinese; (4)reporting of complete response (CR) rate, partial response (PR) rate, overall response (OR) rate, ED rate, or other toxicity data. The extracted data included publication date, regimen used, age, gender, number of patients with CR and OR data, number of patients with data on major adverse events including ED, myelosuppression, infection and haemorrhage.

### Statistical Analysis

The CR rates of patients treated with HAG regimen were directly extracted from individual studies. For subgroup analysis of patients with newly diagnosed AML, refractory/relapsed AML, elderly AML patients (≥60 years) or advanced MDS patients, numbers of patients with CRs were extracted from individual studies and CR rates were recalculated from the derived data. For studies with a control group, the odds ratio (OR) was used for CR rates and adverse events rates. We assessed heterogeneity in the results of the trials using the χ^2^ test of heterogeneity and the I^2^ measure of inconsistency. We considered that heterogeneity was present when the *P* value of the Cochran Q test was <0.05 and the I^2^ statistic was >50%. The random effect model was used for meta-analysis if there was significant heterogeneity and the fixed effect model was used when heterogeneity was not significant. A statistical test with a *P* value less than 0.05 was considered significant. All statistical analyses were performed using the Meta-Analysis program of STATA software (version 12.0 for Windows; Stata Corp LP, College Station, USA).

## Results

### Study Selection

**Fig 1:**
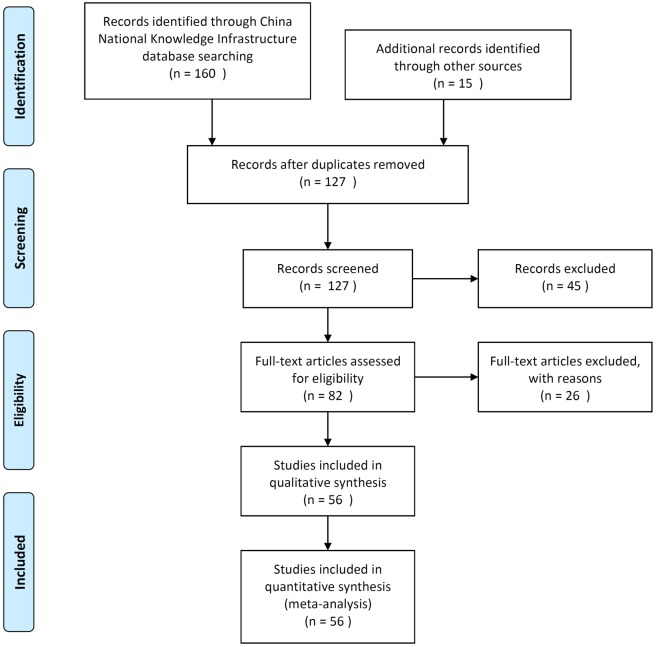
Quorum flow chart of study inclusion. Illustration of the number of articles identified in the literature search and reasons for exclusion.

The search strategy identified 175 records that were screened for inclusion. 48 studies were excluded on ground of duplicated or overlapping reporting. Based on title and abstract review, a total of 45 studies were determined to be inapplicable to HAG regimen trials, and were excluded. Additionally, we excluded 26 studies based on an insufficient number of study participants or case reporting. In total, 56 trials involving 2,314 patients performed between 2006 and 2014 fulfilled the inclusion criteria (Fig 1).

### Study Characteristics

Characteristics of the 56 trials are listed in Tables 1–3. Of the 56 publications included in the meta-analysis, 41 trials with 1,601 patients focused on AML and 11 trials with 536 patients focused on MDS/transformed AML (MDS/t-AML). The remaining four studies [11–14] enrolled both AML and MDS/t-AML patients and were separated into AML and MDS/t-AML groups for meta-analysis. In three of these four trials [12–14], the number of MDS patients was less than 20 and the MDS patients from these studies were excluded from meta-analysis. The HAG regimen was compared to intensive chemotherapy in 16 controlled trials with 736 patients and to the CAG regimen in 10 controlled trials with 490 patients. The number of patients ranged from 20 to 97. The median age of the patients ranged from 39 to 72 years of age, with 40% to 83% male subjects amongst the studies reporting gender. Three studies [15–17] did not indicate the age range. The HAG regimen consisted of low-dose HHT 1–2 mg/m^2^, IV, QD on days 1–14, low-dose Ara-C 10 mg/m^2^, SQ Q12 hr on days 1–14 and G-CSF 200 μg/m^2^, SQ QD on days 1–14 in most studies. A total of 1,754 AML patients were included in 45 studies. Among the 1,754 AML patients, 318 patients were newly diagnosed with AML, 433 were relapsed/refractory (R/R) AML patients and 536 were elderly (≥60 years) AML patients. Among the 536 elderly patients, 238 patients were newly diagnosed with AML. According to International Prognostic Scoring System (IPSS) scores, 207 patients in five trials were considered to have intermediate-2 or high risk MDS. For response data, all trials applied International Working Group (IWG) 2000 response criteria or custom criteria which were similar to IWG 2000. For adverse events data, 25 trials applied World Health Organization grade criteria and the remaining 24 trials did not indicate the adverse events grade.

**Table 1:** Clinical information of trials using HAG regimen in this study.

Study	Type of Disease	Patients (No.)	Median age (range)	Cytarabine dosage	Homoharringtonine dosage	Efficacy			Early death (%)	Toxicity		
										Myelo-suppression (%)	Infection (%)	Haemorrhage (%)
						CR (%)	PR (%)	OR (%)				
**Liu**[35]	AML (ND)	31	63 (57–72)	10mg/m2/12h×14d	1mg/m2/d×14d	58	23	81	3.23	NR	32.26	32.26
**Wei**[36]	AML (R/R)	20	39 (10–62)	10mg/m2/12h×14d	1-2mg/d×14d	70	10	80	NR	60.00	45.00	NR
**Li**[37]	AML (Elderly)	21	66 (60–72)	7.5mg/m2/12h×14d	1mg/m2/d×14d	48	19	67	0.00	NR	57.14	NR
**Liao**[38]	AML (R/R)	20	46 (18–78)	10mg/m2/12h×14d	2mg/d×7-10d	40	30	70	NR	NR	30.00	NR
**Chen**[39]	AML (ND & Elderly)	31	69 (60–85)	10mg/m2/12h×14d	1mg/m2/d×14d	74	10	84	0.00	51.61	41.94	NR
**Liu**[40]	AML (Elderly)	25	68 (62–80)	10mg/m2/12h×14d	1-2mg/d×14d	32	40	72	8.00	NR	60.00	NR
**Zhou**[41]	AML (ND)	25	67 (40–83)	10mg/m2/12h×14d	1mg/m2/d×14d	56	16	72	4.00	NR	28.00	NR
**Zhang**[31]	AML (R/R)	46	46 (18–75)	7.5mg/m2/12h ×14d	1.5mg/m2/d×14d	39	11	50	21.74	17.39	28.26	NR
**Ye**[42]	AML (Elderly)	31	68 (60–79)	10mg/m2/12h×14d	1mg/d×14d	74	10	84	0.00	NR	41.94	48.39
**Chen**[11]	AML (R/R)	79	49 (32–82)	10mg/m2/12h×14d	1mg/m2/d×14d	43	18	61	0.00	49.00	52.00	NR
**Zhang**[43]	AML (ND & Elderly)	23	71 (60–82)	10mg/m2/12h×10-15d	1mg/d×10-15d	52	22	74	8.70	NR	NR	NR
**Xu**[12]	AML (ND+R/R)	18+13	52 (15–81)	15-25mg/m2/12h×14d	1mg/m2/d×14d	48	NR	NR	0.00	NR	45.00	37.50
**Ma**[15]	AML (R/R)	68	NR	10mg/m2/12h×14d	1mg/m2/d×14d	44	19	63	0.00	60.29	38.24	0.00
**Yang**[44]	AML	28	NR (21–88)	25-50mg/d×14d	1mg/d×14d	54	25	79	NR	NR	NR	NR
**Sun**[45]	AML (Elderly)	18	NR (63–83)	10mg/m2/12h×14d	1-2mg/d×14d	78	11	89	5.56	NR	NR	NR
**Tong**[46]	AML (Elderly)	25	NR (60–78)	10mg/m2/12h×14d	1mg/m2/d×14d	32	20	52	4.00	NR	72.00	NR
**Li**[47]	AML (R/R)	20	39 (22–65)	10mg/m2/12h×14d	1mg/m2/d×14d	70	15	85	NR	65.00	NR	NR
**Yi**[48]	AML	24	NR (54–79)	10mg/m2/12h×14d	1-2mg/d×14d	42	50	92	5.00	NR	100.00	40.00
**Yang**[49]	AML (Elderly)	20	68 (60–73)	10mg/m2/12h×14d	1mg/d×14d	45	30	75	NR	NR	NR	NR
**Zhan**[50]	AML (Elderly)	14	70 (60–81)	10mg/m2/12h×14d	1mg/m2/d×14d	50	14	64	0.00	NR	NR	NR
**Xie**[51]	AML (ND & Elderly)	20	69 (61–80)	10mg/m2/12h×14d	1mg/m2/d×14d	55	10	65	10.00	65.00	70.00	55.00
**Huang**[52]	AML	20	63 (55–83)	10mg/m2/12h×14d	1mg/m2/d×14d	70	15	85	0.00	80.00	30.00	0.00
**Guan**[53]	AML (Elderly)	29	64 (60–81)	10-15mg/m2/12h×14d	1-2mg/d×8d	41	17	59	3.45	51.72	41.38	51.72
**Cheng**[54]	AML (ND & Elderly)	28	67 (60–88)	10mg/m2/12h×14d	1mg/m2/d×14d	68	14	82	0.00	28.57	35.71	35.71
**Guo**[16]	AML (R/R)	39	NR	10mg/m2/12h×14d	1mg/m2/d×14d	62	8	69	0.00	69.23	66.67	NR
**Ji**[55]	AML	37	45 (15–71)	10mg/m2/12h×14d	1mg/m2/d×14d	46	16	62	NR	45.95	43.24	0.00
**Sun**[56]	AML (Elderly)	14	NR (62–75)	10mg/m2/12h×14d	1mg/m2/d×14d	64	14	79	7.14	NR	NR	NR
**Zheng**[57]	AML (ND)	19	NR (30–74)	25mg/12h×14d	1mg/d×14d	68	NR	NR	10.53	NR	NR	NR
**Wu**[58]	AML (R/R)	21	46 (19–72)	10mg/m2/12h×14d	1mg/m2/d×14d	24	38	62	NR	9.52	33.33	NR
**Han**[59]	AML (ND)	28	NR (36–68)	10mg/m2/12h×14d	1mg/m2/d×14d	71	11	82	3.57	NR	78.57	53.57
**Wang**[13]	AML (Elderly)	21	66 (60–81)	10mg/m2/12h×14d	1mg/m2/d×14d	48	19	67	0.00	73.33	63.33	0.78
**Sun**[14]	AML	22	50 (35–70)	15mg/m2/12h×14d	1mg/d×14d	27	9	36	3.57	NR	42.86	21.43
**Gu**[60]	AML (R/R)	67	50 (15–77)	7.5mg/m2/12h×14d	1.5mg/m2/d×8d	52	12	64	10.45	NR	86.57	NR
**Zhou**[61]	AML (Elderly)	20	67 (60–81)	10mg/m2/12h×14d	1mg/m2/d×14d	50	25	75	5.00	NR	NR	NR
**Zhang**[62]	AML (Elderly)	36	71 (60–80)	10mg/m2/12h×14d	1mg/m2/d×14d	72	6	78	0.00	NR	33.33	NR
**Li**[63]	AML (R/R)	20	42 (22–62)	10mg/m2/12h×14d	1.5mg/m2/d×14d	65	15	80	0.00	60.00	40.00	0.00
**Guo**[64]	AML	16	NR (46–78)	10-20mg/m2/12h×14d	1-2mg/d×14d	50	44	94	0.00	NR	NR	NR
**Li**[65]	AML (ND & Elderly)	21	68 (61–79)	10mg/m2/12h×14d	1mg/m2/d×14d	43	19	62	9.52	85.71	76.19	100.00
**Zhang**[17]	AML	38	67	10mg/m2/12h×14d	1mg/d×14d	66	16	82	0.00	NR	13.16	NR
**Zhang**[66]	AML (ND)	23	40 (35–84)	10mg/m2/d×14d	2mg/d×14d	65	9	74	8.70	NR	82.61	69.57
**Zhang**[67]	AML (ND & Elderly)	25	68 (>60)	10mg/m2/12h×14d	2mg/m2/d×10-14d	52	16	68	12.00	68.00	76.00	NR
**Chen**[30]	AML (ND & Elderly)	56	72 (60–80)	10mg/m2/12h×14d	1.5mg/m2/d×14d	61	14	75	7.14	NR	82.14	NR
**Su**[68]	AML (Elderly)	38	NR (60–78)	10mg/m2/12h×14d	1mg/m2/d×14d	37	37	74	NR	NR	21.05	NR
**Jia**[69]	AML (R/R)	20	41 (18–58)	10-15mg/m2/12h×14d	1mg/m2/d×14d	60	25	85	NR	NR	45.00	NR
**Long**[70]	AML (Elderly)	20	70 (66–77)	10-12mg/m2/12h×14d	1mg/m2/d×14d	40	20	60	NR	15.00	25.00	20.00
**Shu**[71]	MDS/t-AML	28	52 (40–65)	10mg/m2/12h×14d	1-2mg/d×14d	54	21	75	0.00	60.71	46.43	NR
**Yuan**[72]	MDS/t-AML (advanced)	21	57 (31–79)	10mg/m2/12h×14d	1mg/m2/d×14d	29	29	57	0.00	NR	NR	NR
**Chen**[11]	MDS/t-AML	21	56 (46–70)	10mg/m2/12h×14d	1mg/m2/d×14d	NR	NR	52				
**Wu**[73]	MDS/t-AML	32	68 (17–88)	25mg/d×14d	1mg/d×14d	47	25	72	0.00	37.50	NR	NR
**Su**[74]	MDS/t-AML	30	68 (17–88)	25mg/d×14d	1mg/d×14d	47	23	70	0.00	40.00	NR	NR
**Li**[75]	MDS/t-AML	23	60 (16–78)	10-20mg/m2/d×14d	0.5-1mg/m2/d×10-14d	48	26	74	0.00	NR	47.83	8.70
**Sun**[76]	MDS/t-AML (advanced)	16	NR (46–78)	10-20mg/m2/12h ×14d	1-2mg/d×14d	44	38	81	0.00	NR	NR	NR
**Wu[32]**	MDS/t-AML (advanced)	33	71 (60–88)	25mg/d×14d	1mg/d×14d	58	9	67	0.00	NR	NR	NR
**Liu**[77]	MDS/t-AML (advanced)	97	54 (18–84)	20mg/12h×14d	2mg/d×8d	15	48	63	9.28	NR	41.24	NR
**Li**[78]	MDS/t-AML	16	55 (33–78)	10mg/m2/12h×14d	2mg/d×14d	56	31	88	0.00	NR	NR	NR
**Zhang**[79]	MDS/t-AML	20	45 (32–65)	10mg/m2/12h×14d	2mg/d×14d	65	15	80	NR	NR	NR	NR
**Deng**[80]	MDS/t-AML (advanced)	40	57 (47–82)	10mg/m2/d×14d	1mg/m2/d×14d	40	33	73	NR	NR	NR	NR

Abbreviations: CR = complete response; PR = partial response; OR = overall response; ND = newly diagnosed; R/R = relapsed/refractory; advanced = intermediate-2 or high risk; NR = not reported.

**Table 2:** Clinical information of trials using intensive chemotherapy in this study.

Study	Patients (NO.)	Type of Disease	Median age (range)	Regimen	Dosage	CR (%)	ED (%)	Myelo-suppression (%)
					Cytarabine (range of dose)			
					100-200mg/m2/d×7d			
**Sun**[34]	18	AML (Elderly)	NR (61–82)	HA	HHT 1-2mg/d×14d	38.89	22.2	100
**Sun**[46]	12	AML (Elderly)	NR (64–72)	HA	HHT 3-4mg/d×7d	58.33	16.7	NR
**Ye**[28]	25	AML (Elderly)	68 (60–77)	DA	DNR 40mg/d×3d	44	16	76
**Guo**[56]	16	AML	NR (45–76)	HA	HHT 1-2mg/d×14d	18.75	43.7	100
**Sun**[70]	16	MDS/t-AML (advanced)	NR (45–76)	HA	HHT 1-2mg/d×14d	18.75	43.7	100
**Zhang**[74]	20	MDS/t-AML	45 (32–75)	HA	HHT 3-4mg/m2/d×7d	50	NR	NR
**Zheng**[47]	16	AML (ND)	NR (30–47)	HA/DA	HHT 3-4mg/m2/d×7d	37.50	18.7	31.2
					DNR 45mg/m2/d×3d			
**Tong**[35]	27	AML (Elderly)	NR (60–76)	DA	DNR 40mg/m2/d×3d	33.33	11.1	NR
**Liu**[20]	34	AML (ND)	62 (54–68)	HA	HHT 4mg/d×7d	32.35	8.82	82.3
**Jia**[63]	15	AML (R/R)	36 (15–41)	DA	DNR 45mg/m2/d×4-5d	46.67	NR	NR
**Yang**[33]	21	AML (Elderly)	68 (60–73)	DA/HA/MA	DNR 40mg/m2/d×3d	33.33	28.5	NR
					HHT 3-4mg/d×5-7d			
					MITX 5-10mg/m2/d×3d			
**Su**[68]	33	MDS/t-AML	65 (47–85)	HA	HHT 2-3mg/d×7d	33.33	0	NR
**Wu**[48]	21	AML (R/R)	44 (18–70)	MAE	MITX 10mg/m2/d×3d	14.29	NR	66.6
					VP-16 60mg/m2/d×5d			
**Deng**[75]	45	MDS/t-AML (advanced)	58 (38–76)	HA	HHT 1mg/m2/d×14d	17.78	NR	73.3
**Zhou**[53]	18	AML (Elderly)	67 (60–81)	MA	MITX 4-6mg/m2/d×3d	38.89	11.1	NR
**Zhang**[54]	22	AML (Elderly)	NR (61–82)	HA	HHT 1mg/m2/d×14d	36.36	0	NR

Abbreviations: CR = Complete response; ED = earlydeath; ND = newly diagnosed; R/R = relapsed/refractory; advanced = intermediate-2 or high risk; NR = not reported; HA = homoharringtonine (HHT) and cytarabine; DA = daunorubicin (DNR) and cytarabine; MA = mitoxantrone(MITX) and cytarabine; MAE = mitoxantrone (MITX), cytarabine and etoposide (VP-16).

**Table 3:** Clinical information of trials using CAG regimen in this study.

Study	Patients (NO.)	Type of Disease	Median age (range)	Cytarabine dosage	Aclacinomycin dosage	CR (%)
**Li**[78]	12	MDS	53 (40–76)	10mg/m2/12h×14d	14mg/m2/d×4d	50
**Su**[68]	52	AML (Elderly)	NR	10mg/m2/12h×14d	10-14mg/m2/d×4d	73.08
**Li**[65]	22	AML (Elderly)	68 (61–79)	10mg/m2/12h×14d	10-14mg/m2/d×4d	54.55
**Li**[47]	18	AML (R/R)	38 (19–65)	10mg/m2/12h×14d	5-7mg/m2/d×8d	72.22
**Chen**[39]	34	AML (ND & Elderly)	69.5 (60–85)	10mg/m2/12h×14d	14mg/m2/d×8d	67.65
**Wei**[36]	20	AML (R/R)	38 (12–66)	10mg/m2/12h×14-28d	20mg/d×4d	75
**Yi**[48]	24	AML	NR (54–79)	10mg/m2/12h×14d	10mg/m2/d×7d	25
**Su**[74]	33	MDS/t-AML	60 (28–77)	25mg/d×14d	10mg/d×8d	42.42
**Long**[70]	20	AML (Elderly)	70 (60–81)	25mg/m2/12h×14d	9mg/m2/d×7d	65
**Zhan**[50]	21	AML (Elderly)	68 (60–77)	10mg/m2/12h×14d	14mg/m2/d×4d	66.67

Abbreviations: CR = Complete response; ND = newly diagnosed; R/R = relapsed/refractory; NR = not reported.

### Results of Meta-analysis

#### Efficacy of the HAG regimen in all AML and MDS/transformed AML patients

**Fig 2:**
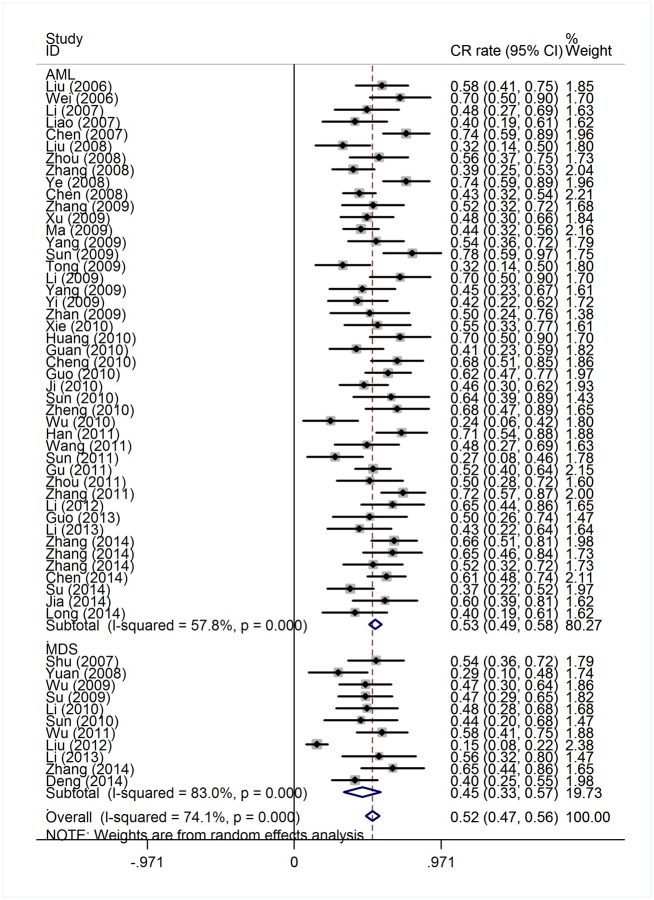
Complete response rates of AML and MDS patients treated with HAG.

**Fig 3:**
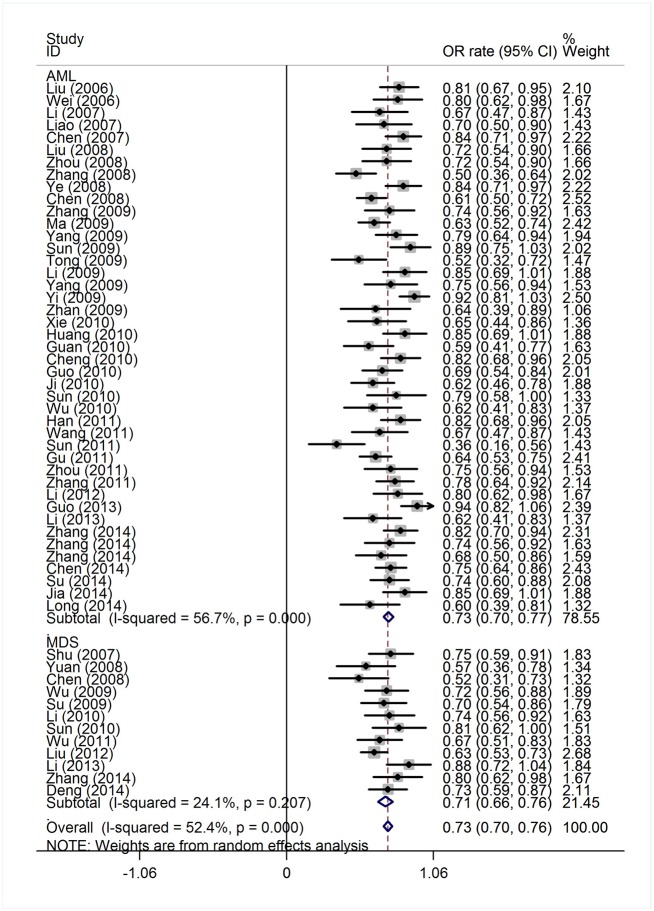
Overall response rates of AML and MDS patients treated with HAG.

The efficacy end points were CR and OR rates. The response outcomes of the HAG regimen in AML patients and MDS/t-AML patients are listed in Table 1. The heterogeneity test of CR event rates of all AML and MDS/t-AML patients from the 56 studies revealed a Cochran Q test *P* value of 0.000 and I^2^ of 74.1%, indicating high heterogeneity. Therefore, the CR event rates were calculated using the random-effects model (Fig 2). The overall CR rate for the 1,654 patients was 52% (95% CI, 47%-56%). Data available from 45 trials with 1,298 AML patients showed that the CR rate was 53% (95% CI, 49%-58%). In the 356 MDS/t-AML patients from 11 trials, the CR rate was 45% (95% CI, 33%-57%). The higher CR rate in AML patients compared to MDS/t-AML patients was statistically significant (*P* = 0.007). There were 54 trials available for pooled estimate of OR event rates. The heterogeneity test of OR event rates revealed a Cochran Q test *P* value of 0.000 and I^2^ of 52.4%, indicating high heterogeneity. To determine the OR rate with high heterogeneity, we used a random-effects model (Fig 3). The OR rate for the 1,625 patients was 73% (95% CI, 70%-76%). For the 1,248 AML patients from 43 trials, the OR rate was 73% (95% CI, 70%-77%). Data available from 12 trials with 377 MDS/t-AML patients showed an OR rate of 71% (95% CI, 66%-76%). There was no significant difference in OR rates between the AML and MDS/t-AML patients (*P* = 0.467).

#### Efficacy of the HAG regimen in newly diagnosed versus relapsed/refractory AML patients

**Fig 4:**
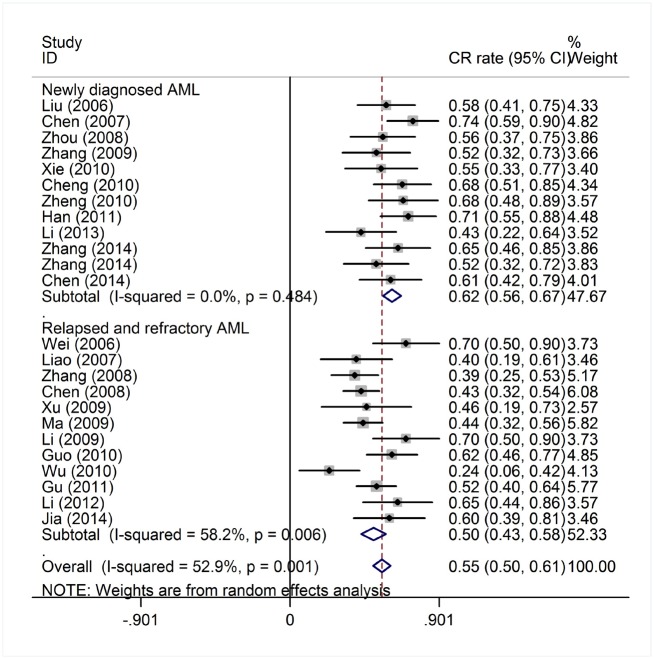
Complete response rate of patients with newly diagnosed AML and relapsed/refractory AML treated with HAG.

There were a total of 318 newly diagnosed AML patients from 12 studies and 433 R/R AML patients from 12 studies (Table 1, Fig 4). Another 21 studies did not specify the AML status of patients and were therefore excluded for this comparison. The heterogeneity test of CR event rates of these 24 studies revealed a Cochran Q test *P* value of 0.001 and I^2^ of 52.9%, indicating high variability. Therefore, the CR event rates were calculated using the random-effects model. The proportion of patients with CR was significantly higher in newly diagnosed AML patients (62%, 95% CI, 56%-67%) than in R/R AML patients (50%, 95% CI, 43%-58%; *P* = 0.001).

#### Efficacy of the HAG regimen in elderly AML and advanced MDS patients

**Fig 5:**
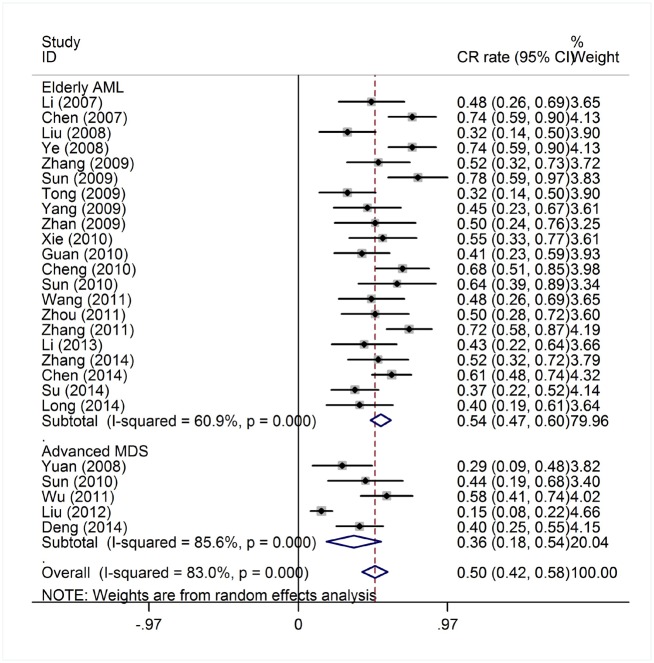
Complete response rate of elderly AML and advanced MDS patients treated with HAG.

There were a total of 536 elderly AML patients (≥60 years) from 21 studies and 207 patients with advanced MDS (intermediate-2 or high risk) from five studies (Table 1). The heterogeneity test of CR event rates of these studies revealed a Cochran Q test *P* value of 0.000 and I^2^ of 83%. Due to the high heterogeneity, the CR event rates were calculated using a random-effects model (Fig 5). The CR rates were 54% (95% CI, 47%-60%) in elderly AML patients and 38% (95% CI, 18%-54%) in advanced MDS patients. There was no significant difference in CR rates between elderly AML patients and all AML patients (*P* = 0.721), suggesting that the HAG regimen was also effective in elderly patients.

#### HAG regimen versus intensive chemotherapy for AML/MDS induction therapy

**Fig 6:**
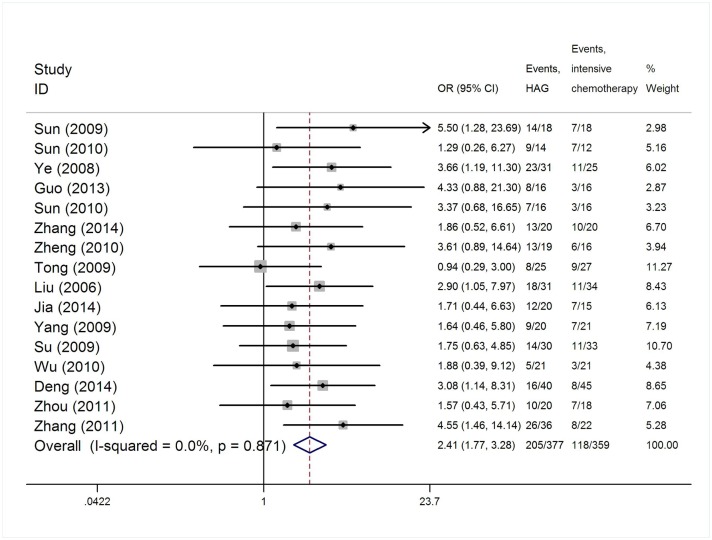
HAG regimen versus intensive chemotherapy for AML/MDS induction.

A comparison of the HAG regimen and intensive chemotherapy for AML/MDS induction therapy was performed using historical controls in a total of 16 trials. There were a total of 307elderly AML patients (≥60 years) from seven studies, 77 R/R AML patients from two studies and 117 patients with advanced MDS (intermediate-2 or high risk) from two studies. Three hundred and seventy-seven patients were treated with HAG and 359 patients were induced with intensive chemotherapy including HA (HHT and cytarabine), DA (daunorubicin and cytarabine), MA (mitoxantrone and cytarabine) and MAE (mitoxantrone, cytarabine and etoposide; regimen details are provided in Table 2). The heterogeneity test of CR event rates from the 16 studies revealed a Cochran Q test *P* value of 0.871 and I^2^ of 0%, indicating that there was no significant variation among the 16 studies. Therefore, the CR event rates were calculated using the fixed-effects model (Fig 6). The CR rate was 55% (95% CI,46%-63%) in patients treated with the HAG regimen. Among them, the CR rates were 60% in elderly AML patients, 39% in R/R AML patients and 41% in advanced MDS patients. The CR rate for patients treated with intensive chemotherapy was only 30% (95% CI, 26%-35%). Among them, the CR rates were 39% in elderly AML patients, 18% in R/R AML patients and 29% in advanced MDS patients. Using historical controls, the CR rates of HAG-treated patients were significantly higher than those of standard induction regimens, with an odds ratio of 2.41 (95% CI, 1.77–3.28; *P* = 0.000).

#### HAG versus CAG for AML/MDS induction therapy

**Fig 7:**
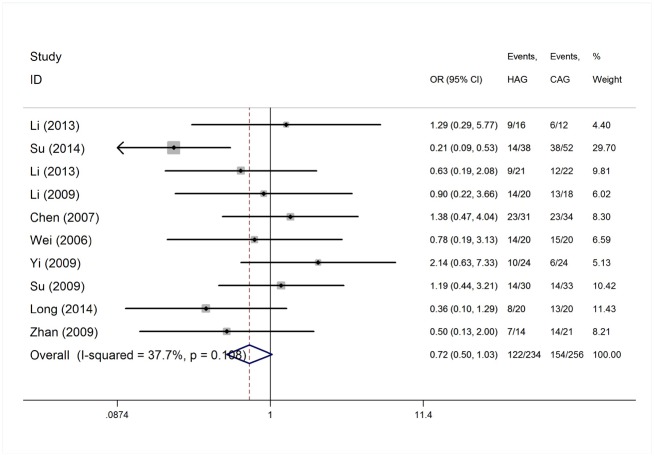
HAG versus CAG therapy for AML/MDS induction.

The CAG regimen has been widely used in China and Japan and has proven effective and safe for the treatment of AML and MDS patients. Using historical controls, the HAG regimen was compared with CAG for AML/MDS induction therapy in ten trials. Two hundred and thirty-four patients were treated with the HAG regimen and 256 patients were induced with the CAG regimen (Table 3). The heterogeneity test of CR event rates from the ten studies revealed a Cochran Q test *P* value of 0.108 and I^2^ of 37.7%, indicating low heterogeneity amongst the studies. Therefore, the CR event rates were calculated using the fixed-effects model (Fig 7). No significant difference was observed between the HAG and CAG-treated groups with an odds ratio of 0.72 (95% CI, 0.50–1.03; *P* = 0.073; Fig 7).

#### Early death rate and hematological toxicity of the HAG regimen

**Fig 8:**
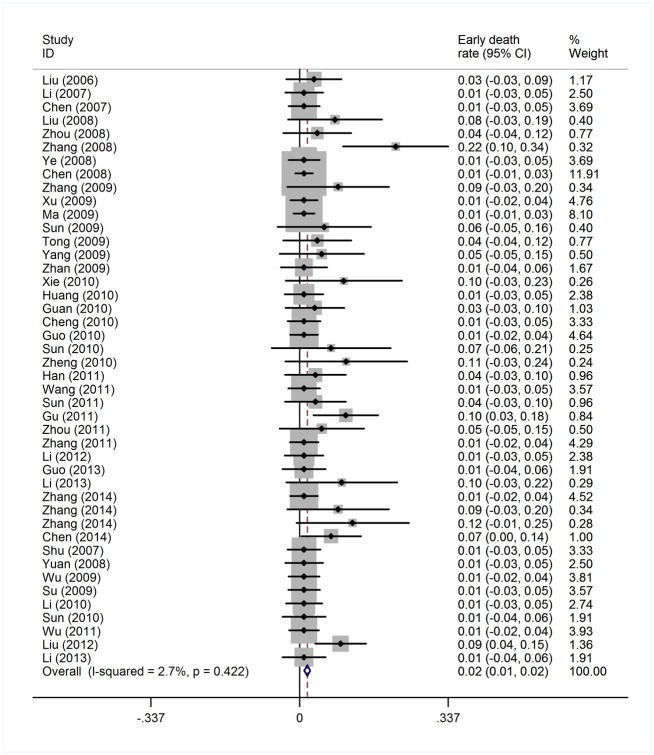
Early death rate in patients treated with the HAG regimen.

**Fig 9:**
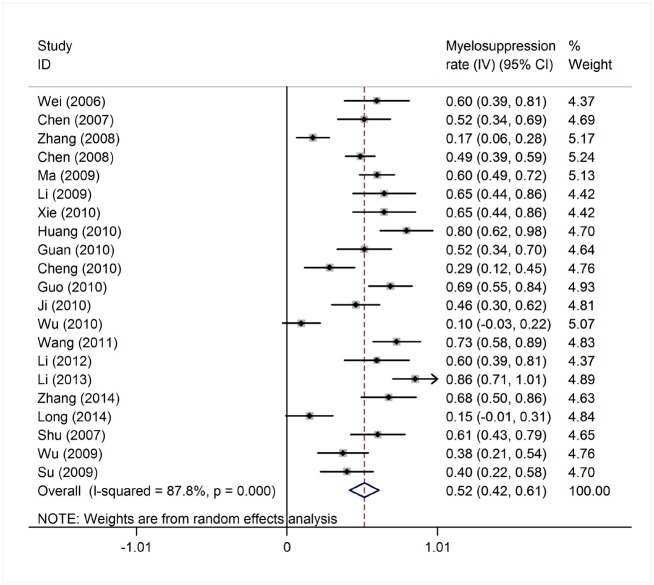
Grade IV myelosuppression rate in patients treated with the HAG regimen.

**Fig 10:**
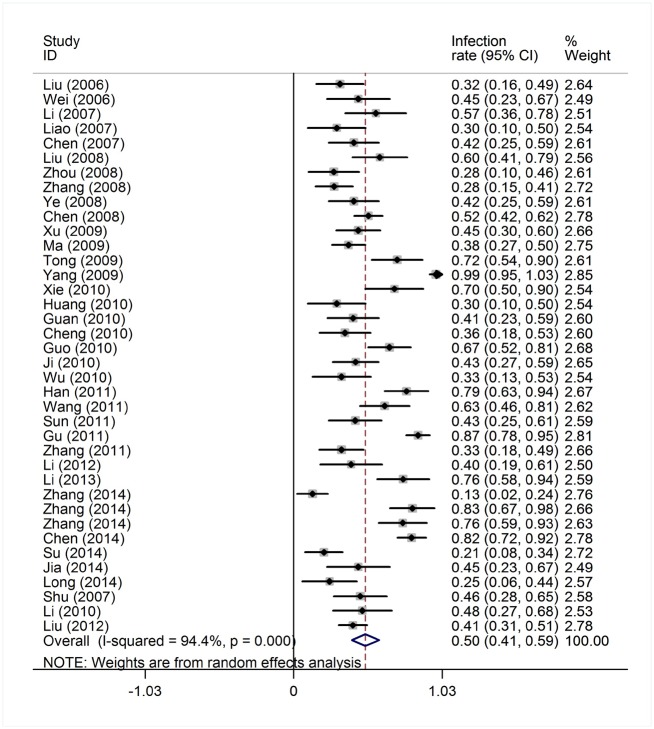
Infection rate in patients treated with the HAG regimen.

**Fig 11:**
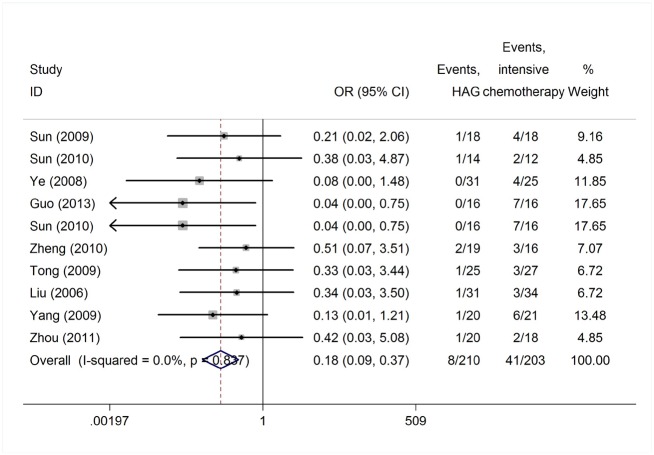
Early death rates in patients treated with the HAG regimen vs. intensive chemotherapy.

**Fig 12:**
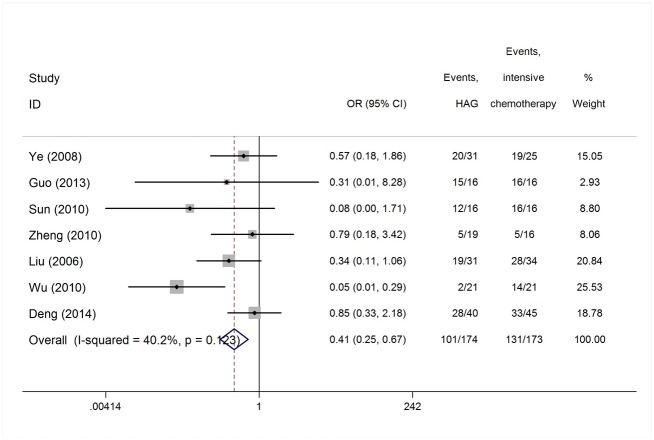
Rates of myelosuppression in patients treated with the HAG regimen vs. intensive chemotherapy.

Reported toxicities in the majority of studies included myelosuppression, infection, nausea and vomiting. ED was reported in 44 studies of 1,391 patients, grade IV myelosuppression data was reported in 21 studies with 350 patients and infection data was reported in 38 studies with 645 patients (Table 1). The heterogeneity test of ED rates revealed a Cochran Q test *P* value of 0.422 and I^2^ of 2.7%, indicating low heterogeneity. Therefore, the ED event rates were calculated using the fixed-effects model. The pooled estimate of ED rates was 2% (95% CI, 1%-2%; actuarial rate 3.95%, 55/1391, Fig 8). Due to the high heterogeneity, the grade IV myelosuppression and infection events rates were calculated using the random-effects model. The pooled estimates of grade IV myelosuppression rate and infection rate were 52% (95% CI, 42%-61%) and 50% (95% CI, 41%-59%), respectively (Figs 9 and 10). When compared with intensive chemotherapy, HAG-treated patients had lower ED and myelosuppression event rates with an odds ratio of 0.18 (95% CI, 0.09–0.37; *P* = 0.000) and 0.41 (95% CI, 0.25–0.67; *P* = 0.000), respectively (Figs 11 and 12).

## Discussion

For the past two decades the CAG regimen has been applied in clinical practice. Based on a meta-analysis 35 studies including 1,029 AML and MDS patients [3], treatment of AML patients with CAG achieved a CR rate of up to 57.9%, a rate which was significantly higher than was observed following treatment with non-CAG regimens. A comparison of CR rates between newly diagnosed AML patients (56.7%) and R/R AML patients (60.1%) revealed no significant difference, indicating that the regimen is effective in the treatment of AML, including for R/R patients. Unfortunately, repeated administration of anthracyclines is associated with dose-dependent toxicity in cardiac myocytes and interstitial damage that can result in early diastolic and later systolic cardiac impairment. Compared with anthracyclines, HHT has a similar therapeutic effect with milder cardiac toxicity. Moreover, HHT is cheap and can be easily accepted by the majority of patients in China.

Studies on HHT have demonstrated efficacy in both *in vitro* and *in vivo* models, with treatment inhibiting the formation of leukemic clones by 50%. Mechanistically, HHT induces apoptosis of leukemia cells through incorporation into the DNA of the cells and inhibition of DNA synthesis [18]. HHT prevents binding of the aminoacyl-tRNAs and RNA substrate to the 60S ribosomal subunit, precluding peptide bond formation and inhibiting the elongation phase of translation [19]. HHT-induced apoptosis in leukemia cells is also associated with down-regulation of telomerase [20] and decreased expression of the inhibitors of apoptosis (IAP) gene family member survivin [21].

The low-dose regimen of HAG effectively reduces the number of myeloblasts, improves pancytopenia, and induces remission notonly in high-risk MDS patients but also in R/R AML, especially for elderly patients. Studies have shown that HHT and cytarabine with the concurrent administration of G-CSF has a potentially synergistic effect in leukemic blasts. In *vivo* G-CSF leads to an enrichment of S-phase leukemic blasts, thereby improving the efficacy of S phase-active chemotherapeutic agents such as cytarabine [10] and significantly reducing the half-killing concentration of cytarabine [22]. G-CSF also enhances the anti-leukemia effect of HHT in leukemic cells by mobilizing resting G0 phase cells into G1 phase. It is shown that CXCR4, a receptor for CXC chemokine receptor 12 (CXCL12), is a central player in migration and homing to tissue niches of leukemia cell that support cell survival, growth and drug resistance[23]; and has proved to be an adverse prognostic indicator of AML[24]. G-CSF can increase the expression of transcriptional repressor growth factorindependence-1 (Gfi-1), which binds to DNA sequences upstream of the CXCR4 gene and inhibits CXCR4 expression in myeloid cells [25].G-CSF also decreases CXCL12 expression by inhibiting activity of osteoblast in the bone marrow [26]. In addition, G-CSF enhances the therapeutic effects of cytarabine and HHT on leukemic cells by promoting the release of granulocytic lineage cells from the bone marrow to the peripheral blood.

This is the first meta-analysis of studies examining the effectiveness of AML and MDS treatment with the HAG regimen. In this meta-analysis, 2,314 patients with AML and MDS were included. The overall CR rate of the HAG regimen was as high as 53% in AML patients and 45% in MDS/t-AML patients. No significant differences in CR rates were observed between all AML patients and elderly AML patients, suggesting that the HAG regimen is also effective in elderly patients. A potential explanation for this result could be that the application of low-dose chemotherapy extends the duration of drug action, increases the cumulative drug dosage and is better tolerated by elderly patients in poor physical condition due to its relatively mild toxicity. The CR rate observed in HAG-treated patients (62%) with newly diagnosed AML is close to that observed with a standard “3+7” regimen (daunorubicin 45 mg/m^2^ and cytarabine 100 mg-200 mg/m^2^;57%-65%) [27–29], suggesting that that HAG may be as effective as the “3+7” regimen. In R/R AML patients, the CR rate was considerably lower than in newly diagnosed AML patients, but still as high as 50%, indicating that the HAG regimen can overcome drug resistance of R/R AML to a certain extent. Unfortunately, due to the high heterogeneity of data in the included studies we could not obtain pooled CR rates of AML patients with different karyotypes or predictive gene mutations such as NPM1, FLT3-ITD and CEBPA. Only six studies reported survival data for patients treated with the HAG regimen. Among them, one study conducted by Chen and colleagues [30] reported the median OS of newly diagnosed AML patients was 12.0±1.7 months with HAG treatment and OS was significantly correlated with age, initial karyotype, PS and gene mutations (NPM1, FLT-ITD and DNMT3A) at diagnosis. With R/R AML patients, the median OS was reported as 12.4 months [31]. The median OS of MDS/t-AML patients was reported as 15.0 months when treated with the HAG regimen and patients with normal serum lactate dehydrogenase (LDH) appeared to have longer median OS when compared to patients with high LDH levels [32]. In comparison of the HAG regimen and intensive chemotherapy for AML/MDS induction therapy in our study,more than 50% of included patients were advanced MDS patients, R/R AML patients and elderly patients with AML. It may explain why the CR rateof intensive chemotherapy in our study (30%) is lower than reported CR rate for intensive chemotherapy in adults (50–80%).Our results suggested that this low-dose regimen of HAG remained superior to the intensive regimens based on CR rate, especially for advanced MDS patients, R/R AML patients and elderly patients with AML, which was consistent with finding reported by Wei *et al*. [3] that CAG had an advantage over AA(aclarubicin and Ara-C) regimen in terms of CR rates.These studies, however, were not randomized, and comparisons were performed with historical controls. Prospective randomized studies should be performed to compare the HAG regimen with standard anthracycline plus cytarabine. Our study also showed that the HAG regimen was as effective as the CAG regimen in the treatment of AML and MDS patients. Due to its lower price and milder cardiac toxicity, the HAG regimen appears more favourable than the CAG regimen.

Cardiovascular failure has been reported in 25% of patients treated with HHT at a dose of 5–6 mg/m^2^ QD using a short infusion schedule [33]. Decreasing the dose to 1.5 mg/m^2^ QD, continuously infused for 14 days resulted in minimal toxicity and was well tolerated by patients [34]. The studies included in our meta-analysis were conducted with a dose of HHT of 1–2 mg/m^2^ daily. An overall induction mortality of 15% was reported in 326 newly diagnosed AML patients from the Cancer and Leukemia Group B 8321 study treated with the “3+7” regimen [29]. Analysis from the Swedish Acute Leukemia Registry of all unselected 2,767 AML cases established an overall ED (defined as 30 days from diagnosis) rate of 19%, while the intensive chemotherapy group had an ED rate of 10% [27]. Our study showed that the pooled ED rate of patients treated with the HAG regimen was only 2% (95% CI, 1%-2%; actuarial rate 3.95%, 55/1391) and the most frequently observed adverse effect was mild to moderate myelosuppression. When compared with intensive induction, we found that HAG has fewer ED and myelosuppression event rates. Taken together, the results of induction therapy efficacy and associated toxicities suggest that the HAG regimen was effective and well tolerated.

This study has several limitations. One of the limitations is that most included studies were single center, retrospective trials and the comparisons in those studies were made with historical controls. The majority of the studies were small (only 16 of the 56 studies had 50 or more subjects), which might influence the reliability of results. Another limitation is that our study is based on 56 trials which were all conducted in China. It is not clear whether similar outcomes would be observed in other Asian or Western countries. In addition, the studies were conducted between 2005 and 2014. More recently identified prognostic markers, including FLT3-ITD, CEBPA and NPM1, were not routinely examined in the studies we included in our analysis. Although all the studies used similar HAG regimens, the dosage of cytarabine varied from 7.5 to 25 mg/m^2^/12h. This is likely a factor leading to the high heterogeneity of the observed response rates. HAG was compared with standard induction regimens from historical controls in 16 studies. The intensive regimens also varied including HA, DA, MA and MAE. Finally, due to the lack of a sufficient amount of data, a meta-analysis could not be performed for survival data.

## Conclusions

The present meta-analysis demonstrated that the HAG regimen is efficacious for the treatment of AML and MDS, particularly in elderly AML patients. There are no significant differences between the CAG regimen and HAG regimen with respect to CR rates. The HAG regimen appears to be more effective than intensive chemotherapy with higher CR rate and safer with less ED and myelosuppression rates, especially foradvanced MDS patients, relapsed/refractory AML patients and elderly patients with AML. Due to the lack of randomized controlled trials in our review, further prospective randomized studies to explicitly determine the efficacy and safety of HHT in comparison with intensive chemotherapy are required. Future trials should also focus on additional issues including the role of hypomethylating agents combined with the HAG regimen in the treatment of AML and MDS.

## Supporting Information

S1 Checklist PRISMA Checklist. (DOC)
